# What do Japanese residents learn from treating dying patients? The implications for training in end-of-life care

**DOI:** 10.1186/s12909-017-1029-6

**Published:** 2017-11-13

**Authors:** Kazuko Arai, Takuya Saiki, Rintaro Imafuku, Chihiro Kawakami, Kazuhiko Fujisaki, Yasuyuki Suzuki

**Affiliations:** 1 Medical Education Development Center, Gifu University, 1-1 Yanagido, Gifu, 501-1194 Japan; 2 Nagasaki Public Health Center, 3-6-24 Nagasaki, Totshima City, Tokyo 171-0051 Japan

**Keywords:** Dying patients, End-of-life care, Residents, Emotion, Experiential learning

## Abstract

**Background:**

How medical residents’ experiences with care for dying patients affect their emotional well-being, their learning outcomes, and the formation of their professional identities is not fully understood. We examine residents’ emotional states and learning occurring during the provision of care to dying patients and specifically discuss the impact of providing end-of-life (EOL) care on professional identity formation.

**Methods:**

Semi-structured interviews were conducted with 13 residents who had graduated in the last 3 to 5 years. Thematic theoretical analysis was applied, and key themes were developed based on Kolb’s experiential learning cycle.

**Results:**

Eight key themes emerged from the analysis. The residents experienced dilemmas in confronting the reality of medical uncertainty as well as a disruption of emotional state and self-efficacy. Although the residents felt a sense of helplessness and guilt, they were able to reflect on strategies for handling medical care that focused on patients and that required a truly sincere attitude. They also contemplated the importance of palliative care and communication with patients, patients’ family members and medical staff. Building on these experiences, the residents rebuilt a sense of awareness that allowed them to directly engage with the type of medical care that they are likely to be called upon to perform in the future as the population continues to age.

**Conclusions:**

This study revealed Japanese residents’ perceptions, emotions and learning processes in caring for dying patients by applying Kolb’s experiential learning theory. The findings of this study may illuminate valuable pieces of knowledge for future education in EOL care.

## Background

In an era in which the population is aging, it is indispensable for doctors to respect dying patients and to be able to provide appropriate end-of-life (EOL) care. Training programs must be instituted for physicians to develop these competencies [1–3]. In fact, the concept of EOL/palliative care for physicians and other medical practitioners is giving rise to the spread of basic EOL/palliative care in Japan, developing broad foundations for this type of practice [4]. However, physicians in palliative care specialty training have had markedly unmet needs regarding training on comprehensive contents and education, and therefore, they demand a systematic training program to learn methods of educating students and residents about palliative medicine [5], which indicates that palliative care and its education in Japan are in a period of transition.

In Japan, elderly people over the age of 65 accounted for 26.8% of the population in 2015, and this proportion is expected to increase to more than 30% by 2025 [6]. Because this issue is important not only in Japan but also worldwide, enhanced training in EOL care is increasingly and universally in demand [1–3].

Mason and colleagues indicated that undergraduate palliative care training improved self-efficacy and postgraduate activities, including communication skills, holistic pain management, and team care [7]. Regarding residency education, Schmit and colleagues, through a questionnaire survey, found that residents who reported more classroom training during their residency were more comfortable with EOL conversations. By contrast, a passive learning culture in hospitals has led to a loss of invaluable learning opportunities for EOL care [8]. Incidental, apprentice-style, on-the-job, and opportunistic learning is not an effective strategy for EOL care pedagogy; rather, residents are seeking systematic learning opportunities and high-quality supervision from experts [8]. In fact, many residents often practice EOL care after graduation without any proper training during medical school and their residency [9]. McFarland et al. conducted a survey about significant clinical distress and frustration for internal medicine residents rotating in hematology and oncology. They revealed that attending physicians’ approaches to care for dying patients cause a dilemma for residents who are obligated to carry out the orders of attending physicians or who are unable to understand the plans or prognosis of care [10]. Due to inadequate instruction in EOL care, these residents frequently do not handle this serious situation very well, leading to issues ranging from depressive tendencies to confusion and diminishing resident-patient contact, which in turn negatively impacts the formation of residents’ identities as doctors [11, 12].

To identify residents’ perspectives on this matter, Price and Schofield conducted a qualitative study to identify junior doctors’ key learning experiences and to examine their perceived effectiveness. They demonstrated the influence of the different learning opportunities that are encountered during medical training on residents’ confidence in relation to clinical management and understanding of the role of specialists on a palliative care team [2]. However, the focus of their study was primarily on improving the learning environments of EOL training programs. Since daily workplace experiences are core activities through which residents learn medical care, an investigation of the current status of the actual care that is provided by residents has the potential to become the foundation not only for training in EOL care but also for residents’ identity formation. Specifically, we seek answers to the following questions:

1. What types of psychological/emotional states do residents experience while providing care for dying patients?
2. How do residents learn from caring for dying patients?
3. How do these experiences and learning opportunities contribute to the formation of residents’ professional identity?

## Methods

### Design

We employed a qualitative investigation to build upon the research base of residents’ actual experiences and in-depth psychological/emotional states while providing care for dying patients. The study was designed based on Kolb’s experiential learning theory (ELT) [13]. Kolb’s ELT refers to the role of experience in learning, demonstrating how adults grasp experiences, reflect and transform the learning. Kolb’s experiential learning cycle frames the learning process of active adults and includes four stages: 1) concrete experience, 2) reflective observation, 3) abstract conceptualization and 4) active experimentation [13].

### Participants

The participants were residents in their 3rd to 5th year at a general hospital who had completed 2 years of their junior residency training. Thirteen residents were recruited (8 males, 5 females) using the convenience sampling technique, and these residents agreed to take part in the study. All of them recognized that they had not received any systematic undergraduate education in EOL care. Details regarding the participants are shown in Table 1.

**Table 1:** Participants

Code	Gender	Age ranges	Years of clinical experience
a	M	Thirties	4
b	F	Twenties	4
c	M	Twenties	3
d	M	Thirties	5
e	F	Twenties	4
f	F	Twenties	5
g	M	Twenties	4
h	M	Thirties	3
i	M	Thirties	5
j	F	Thirties	5
k	M	Twenties	5
l	M	Twenties	3
m	F	Forties	4

### Data collection

Semi-structured interviews were conducted to collect data, given that the residents’ demographic characteristics had previously been obtained. In the interviews, we asked the participants to choose the case that had made the deepest impact on them from their experiences with patients who had passed away and to reflect on that experience as deeply as possible. The interview guide was based on Kolb’s ELT [13]. In particular, we attempted to elicit the following information in relation to the residents’ experiences providing care for dying patients: their psychological/emotional and cognitive changes, the interactions between attending physicians and other health professionals, the things that were learned from the case, and the changes that they would make when attending to future EOL cases. The interview guide is shown in Table 2.

**Table 2:** Interview guide

	Question items	Kolb’s ELT
1	Which dying patients left the biggest impression on you? What treatment/care was provided, and how did you feel when you were treating the patient?	Experience / Reflection
2	How did you feel when the patient passed away?	
3	Did you receive advice or support from the attending physician and other medical staff when you were treating the patient?	
4	What do you think will be important when you treat a similar patient in the future?	Conceptualization/Planning
5	How would you like to support residents and students when you become an attending physician?	

*ELT* experiential learning theory

The interviews were individually conducted in Japanese by the first author (KA) in a private room with a safe environment in which it was easy to talk. Each interview lasted approximately 40 to 60 min and was audio recorded using an IC recorder. The recordings were transcribed and then translated into English.

### Data analysis

The thematic theoretical analysis approach [14, 15] was employed to inductively generate and develop salient categories of the residents’ experiences and perceptions of providing care for dying patients. In thematic theoretical analysis, the analysis is driven by the researcher’s theoretical interests and is focused on some specific aspects of the data with the underlying theoretical framework [14]. Kolb’s learning cycle was applied to this labeling process through the residents’ 1) having an experience with a patient who passed away, 2) reflecting on the experience, 3) learning from the experience, and 4) planning or trying out what they learned. It was also assumed that social interactions with attending physicians, other health professionals, patients and patients’ family members could be key to understanding residents’ learning processes more effectively.

KA first entered the transcript into an Excel (Microsoft, Redmond, WA) spreadsheet, and three researchers (KA, TS, and YS) independently categorized the data into Kolb’s experiential learning cycle [13] and then analyzed each data item thematically by hand. The preliminary results of the analysis that was performed by the three researchers were carefully reviewed multiple times by the entire research team, including RI, CK, and KF, to establish the credibility and validity of the data analysis. Data saturation was achieved based on the authors’ agreement after 11 interviews; subsequently, 2 more residents were interviewed to ensure full data saturation.

## Results

Using thematic theoretical analysis, 3 domains and 8 key themes from the residents’ experiences and perceptions were generated (Table 3). Domains 1, 2 and 3 relate to the steps of concrete experiences, reflections, and conceptualization/planning of Kolb’s experiential learning cycle, respectively.

**Table 3:** Residents’ perceptions of caring for dying patients

Theme			Kolb’s ELT
I. Mixed feelings associated with providing care for dying patients			
	1	Dilemmas in confronting the reality of medical uncertainty	Experience
	2	Disrupted self-efficacy due to miscommunication in stressful situations	
	3	Feelings of guilt and powerlessness both as a person and as a doctor	
II. Reflections on the experience of providing care for dying patients			
	1	The importance of facing patients’, families’ and one’s own emotions	Reflection
	2	Broadening of perspectives from individual communication to inter-professional communication	
	3	Renewed awareness from a disease-oriented cure to a palliative care-oriented treatment of pain relief	
III. Rebuilding awareness for EOL care			
	1	A desire for communication that anticipates the future of patients and patients’ families and patient-centered care	Conceptualization/Planning
	2	Self-awareness of professional identity and the desire to continually develop skills	

*EOL* end-of-life

### Mixed feelings associated with providing care for dying patients

During clinical training, the residents had experienced caring for dying patients. Because the residents could not do anything for these patients, they suffered from a guilty conscience and viewed themselves not only as being an incompetent doctor but also as having a useless existence in society. They harbored negative emotions such as anger or irritation with themselves and/or their attending physicians. They also felt a sense of guilt toward the patient and/or the patients’ family. These unresolvable feelings occasionally disrupted the residents’ self-efficacy and made them feel as though the care and treatment that they were providing were incomplete. Conversely, the residents also felt gratitude toward the attending physicians and medical staff members with whom they had established favorable communication or a relationship of mutual trust.

#### Theme I-1. Dilemmas in confronting the reality of medical uncertainty

The residents tended to ask their attending physicians for more concrete methods of caring for patients that fully considered both the biomedical and the psycho-social aspects of the patient. However, the suggested treatment plans given by the attending physicians were often strongly oriented toward the biomedical aspects of disease. Because these suggestions were somewhat different from what they expected, they were puzzled with regard to how to engage in patient care as a health professional.

*"I was grateful for my attending physician, who proposed a variety of medical examinations in general, but in the end, these examinations were focused on cancer and not on the patient as a human being. I feel like I had support with that type of treatment and diagnosis." (e)*

Furthermore, the residents observed the reality where health professionals should manage the uncertain processes of care. For instance, their experiences of problems related to clinical ethics caused many dilemmas for them in regard to the uncertainty of caring for a terminally ill patient.

*"In the informed consent of the blood specialist, after it was determined that the condition was a blood disease, the physician was just waiting on the conflicted family... During this time, the patient's condition worsened. We couldn't make any decisions because the blood specialist's plan was not clear. So, I worried endlessly and wished that they would decide on the plan quickly." (l)*

#### Theme I-2. Disrupted self-efficacy due to miscommunication in stressful situations

When communication did not go well within the medical team, the residents were frequently confused about the lack of consensus regarding treatment. They also experienced difficulties in working on a medical team when they did not receive adequate orientation and when the situation moved more quickly than expected. Attending physicians were frequently absent when the residents were seeking instructions or clarification. Thus, the residents had a sense of dilemma and anxiety, and their self-efficacy was disrupted.

*"The attending physician, I (resident), and the palliative care team were involved. However, it was hard for us to match our pace as a team... So, there was one time when one of the responses was mistaken... I still have strong regrets, and I wish we had adopted an attitude in which we worked more closely together." (g)*

*"Even when I conferred with my attending physician, they said there isn't anything in particular to do because they are terminal, so I couldn't handle my self-doubt about whether there truly wasn't anything that could be done because they were in the EOL stage. So, the patient died while I still had a strong sense of incompletion. I tried to do my very best but..." (i)*

On the other hand, when communication within the medical team was favorable, the attending physicians were a strong source of emotional support for the residents’ participation in the practice. In particular, the residents could feel relaxed and manage their self-efficacy by obtaining support and encouragement from attending physicians who appreciated their sustained effort in training.

*"Both my attending physician and the palliative care physician were nice, and they followed me... I actually got to study, and when I was thinking I would try to do it differently next time so that this type of thing didn’t happen, they steered me and my depressed emotions in a very positive direction. They were very good." (g)*

#### Theme I-3. Feelings of guilt and powerlessness as a person and as a doctor

Some residents distinctly felt inexperienced as a doctor. They felt as though they were placed in a position in which they cared for the sick without being able to do anything. This sense of helplessness and these feelings of guilt seemed to make them want to escape from the actual clinical setting. Moreover, they did not have any means of properly communicating with the patient or the family, and they felt guilt and remorse that they had done something that was irreparable.

*"I felt like I was a very unacceptable human being more than I was an unacceptable doctor. I felt worthless because I couldn't do anything at all for the patient who was right in front of me." (b)*

*"I actually knew it all (the name of the disease and prognosis). But, one time, I avoided it a little bit... It is a difficult thing to do. Even when it is clear that a condition won't get better, there are times when you keep saying get better, get better after starting treatment. You are persuaded by it, and you have to say it." (g)*

### Reflections on the experience of providing care for dying patients

When the residents worked through a difficult situation involving EOL care, such experiences enabled them to expand the scope of their communication with the people surrounding them. By reflecting on tough experiences in caring for dying patients, the residents became aware of the importance of facing patients, families and other health professionals as well as themselves. Their reflections led to a paradigm shift in their concept of EOL care.

#### Theme II-1. The importance of facing patients’, families’ and one’s own emotions

Because patients and their families experience intense emotions under time constraints, the residents tended to adopt evasive behaviors. However, after a certain amount of reflection time, they began to ask themselves what it meant to build a relationship of mutual trust with a patient or a patient’s family, what should be prioritized, and what it meant to support hope. Through these reflections, they attempted to understand the essentials of patient-centered care.

*"The patient got irritated, yelling at the medical staff and his family, and seeing that was really difficult. There was a time when I thought 'I kind of don't want to see him'... I thought I wanted to have a bit more time to talk with the patient. I wondered if I couldn't have worked a bit harder to have the patient get a slightly different understanding of his disease." (e)*

#### Theme II-2. Broadening of perspectives from individual to inter-professional communication

The residents were worried not only about how to convey bad news but also about how they should address the patient’s and the patient’s family’s emotions. Although they reported struggling with such situations, the residents felt that the communication skills or experiences gained through inter-professional cooperation in EOL care as well as reflection after the patient had passed away were useful in terms of managing these worries. The residents also learned the importance of sharing ideas with other staff members and increasing the number of ideas and options for treatment.

*"If I didn't have the reflection at the clinico-pathological conference, I think I would have kept feeling bad or that this unpleasant experience would have kept feeling that way. Because there was an opportunity for reflection, I noticed that I had realizations like 'Oh, this is what I was feeling' or 'I should have done this.' It was amazing and really good. I felt like I will be able to utilize this experience later or maybe that is just my hope." (e)*

*"Unity within the team and setting up as many times for discussions as possible. I think that the attending physicians, residents, palliative care team and the co-medical staff are the same way. I think that having a long time and many settings where opinions can be exchanged is probably important. It is hard to face something by myself, so I think it is important not to forget to approach the patient as a team. Working as a team reduces the burden on the individual and produces ideas. I think these things are important." (g)*

Furthermore, the residents reflected that they shared the mental burden with nurses and obtained positive feedback, encouragement and advice on how to handle specific situations from them. Through interacting with other professionals, such as nurses, the residents could broaden their perspectives on healthcare.

*"There was a nurse who was contradicting what (the doctor) was doing, but another nurse kindly said, 'There was nothing else we could do for the patient,' and helped out together. This somehow made me relax just because there was someone who felt that way." (m)*

#### Theme II-3. Renewed awareness: From a disease-oriented cure to a palliative care-oriented treatment of pain relief

The experience of facing terminally ill or dying patients shifted the residents’ learning from disease-oriented treatment toward palliative care-oriented treatment. When the residents were faced with the patient’s or the patient’s family’s pain, it gave them a new and strong awareness of the necessity of pain relief. It also made them aware of the importance of introducing pro-active palliative care.

*"(Upon asking the palliative care team), I heard that we are (trying to) take away their pain and support what the patient wants to do." (f)*

*"The patient suffered from some severe dyspnea, so, of course, I wanted to properly give her morphine." (h)*

### Rebuilding awareness for EOL care

The residents’ valuable experiences with treating dying patients and facing difficult-to-handle emotions (their own, the patient’s and/or the family’s) made them conceptualize EOL care in their own way such that they could apply it to their next case. They incrementally transitioned into a state in which they constructed their own EOL care method and style.

#### Theme III-1. A desire for communication that anticipates the future of patients and of patients’ families and patient-centered care

The residents became aware of the importance of facing patients and their families directly. They also became aware of the need to exhibit a professional attitude in diagnosing the patient’s condition and to begin treatment early while sensing potential future anxieties. They wanted to practice patient-centered treatment that closely supported the patient’s hopes as much as possible while adequately communicating with the patient and with the family.

*"Naturally, I will ask the patient about their hopes at the beginning. If it is a patient who can see that they will pass away, in the end, I want to prioritize things like letting them die where they want to." (e)*

*"I guess detecting anxiety ahead of time and doing an interview. Being conscious of communication that feels like the other person understood you. I also think it is necessary to give responses while being aware of what type of answer the family is looking for. Of course, it is essential to spend time with families who need time." (i)*

#### Theme III-2. Self-awareness of professional identity and the desire to continually develop skills

The residents reflected on their future in terms of what type of doctor and person they should be based on their various experiences with satisfaction and pain. This was an opportunity for them not only to consider what the image of physicians who work in EOL care should look like but also to take another hard look at their own medical aspirations and the importance of continuing professional development to improve the quality of care. These reflections allowed them to form an image of what learner-centered communication should look like when they become attending physicians.

*"I think that what I want to do as a doctor is to improve the quality of care for the many commonplace illnesses one by one." (i)*

*"(When I become an attending physician), on that day, I will be relaxed and properly explain to the subordinate physicians that I have handled these types of situations in this manner. It's fine, even if it takes me until the end of the day. Then, during reflection, I think (after getting a better understanding) that it should have been a bit different." (b)*

## Discussion

Drawing on Kolb’s ELT [13], this study identified themes in residents’ experiences and awareness in care for dying patients. Experience, which is one of the steps in the learning cycle [13], is positioned as a trigger point that creates new actions based on knowledge of the current situation, information, skills, and cognition. Through this process, this study revealed that the residents gradually reconstructed their awareness of EOL care even while experiencing a variety of negative emotions and disruptions in their sense of self-efficacy. In particular, this study described the changes in the residents’ emotions and perceptions of caring for dying patients as well as their reconceptualization of communication with medical team members, the patient and the patient’s family to provide better EOL care and to form a professional identity as a physician.

Currently, early and systematic provisions of palliative care education in undergraduate education are reported [16, 17]. These interventions will enhance residents’ readiness for EOL care. In postgraduate education, good evidence has supported that residents can develop EOL care competencies [18, 19], although the distress among residents is still increasing in EOL care and training [10]. Therefore, we believe that the findings of our study clearly illuminate valuable pieces of knowledge for future education in EOL care. The implications suggested by this study for attending physicians in EOL care to promote residents’ experiential learning are summarized in Table 4.

**Table 4:** Suggested implications for attending physicians in EOL care to promote residents’ experiential learning

Attending physicians in EOL care should…	
1	Teach residents skills to handle uncertainty, contradictions, and limitations in medicine
2	Establish an explicit learning opportunity that allows residents to communicate with multidisciplinary team members
3	Recognize that they themselves are viewed as role models
4	Pay attention to residents’ emotional status and offer words of affirmation
5	Take various approaches to promoting residents’ deep reflection on their experiences
6	Respect the autonomy of residents and share responsibilities to develop their professional identity

*EOL* end-of-life

The residents’ cognitions and emotions were largely influenced by their experiences of various interactions with attending physicians, nurses, patients and patients’ families, indicating that such conversations were core experiences and the triggers of experiential learning for the residents. Our findings demonstrated residents’ intolerance of uncertainty and their dilemma in response to attending physicians who sometimes planned disease-oriented care. Although the residents asked for instruction regarding medical content from attending physicians with respect to EOL care, this instruction did not necessarily meet their needs. The residents sought suggestions from attending physicians not only concerning the patient’s biomedical status, which changed from minute to minute, but also concerning appropriate coping mechanisms for the family members who were simultaneously watching over the patient. The residents frequently did not receive information from attending physicians that could help them manage such difficult situations. These interactive processes were frequently associated with negative feelings such as anger and frustration both with themselves and with the attending physicians. However, the residents still tended to seek universal and science-based answers, most likely due to the lack of undergraduate education in EOL care [9]. Finally, the residents keenly felt their own inexperience both as human beings and as physicians, resulting in a great deal of stress and confusion due to the highly individual and uncertain nature of EOL care [11, 20].

These findings may be interpreted as cultural characteristics of Japanese individuals. According to Hofstede [21], compared to Western European countries, Japanese people have a stronger tendency to avoid uncertainty and belong to a culture displaying a higher power distance, which is defined as “the degree to which the less powerful members of a society accept and expect that power is distributed unequally” [21]. This hierarchical social system affects the communication behavior of residents (i.e., less powerful members) with their attending physicians during training. Specifically, when Japanese residents had greater anxiety regarding uncertainty in training, it was difficult for them to freely ask for attending physicians’ support to reduce their anxiety. This finding is consistent with previous studies in Japan that have indicated that availability/accessibility is an important but insufficient characteristic of Japanese attending physicians [22]. Attending physicians should teach residents skills to handle uncertainty, contradictions and limitations in medicine through EOL care [23].

Regarding interactions with medical team members, the residents acknowledged the importance of better communication within a medical team, including with nurses, which was one of the most impressive themes concerning which they reflected and learned. A recent study indicated that newly qualified doctors could learn about the importance of calmness and acceptance in EOL care from interdisciplinary role models in nursing homes [24]. Our study demonstrated that residents could learn these topics in the stages of experiential learning, including concrete experiences and reflective observations, even in teaching hospital settings. Specifically, cooperation with nurses was a great opportunity for the residents to learn about respect for other people and health professionals, in addition to the importance of inter-professional collaboration. Therefore, attending physicians should establish an explicit learning opportunity that allows residents to communicate with multidisciplinary team members.

The emotional disruptions of residents in caring for dying patients were newly clarified in this study. Although the residents were inexperienced as doctors, they were regarded as full-fledged doctors by the patients and their families, which might have caused them to struggle in their isolation and may have led them to seek to escape from dying patients. Furthermore, the residents felt apologetic when they witnessed the death of patients, making statements such as “*I really screwed up!”* Such an emotional state is consistent with a previous study on medical students who reportedly felt sadness, inability, fear, anger and anxiety. However, the residents also recognized the limitations of medicine [25], and even had negative emotions from observing the unhelpful modeling of attending physicians, such as disease-oriented care, slow decision-making processes, and non-empathetic comments such as *“nothing to do for this patient.”* By contrast, our study indicates that a limited amount of conversation involving gratitude or support from the supervisor, medical team members, the patient or the patient’s family is a valuable source of psychological support and can help maintain residents’ self-esteem and sense of security. Attending physicians should recognize that they themselves are role models and pay more attention to residents’ emotional status and offer or reinforce words of affirmation for residents.

This study also confirmed that effective reflection is indispensable in helping residents cope with their difficult and negative experiences in caring for dying patients. In the twenty-first century, health professionals are required not only to be technically proficient but also to be reflective [26]. This aspect is considered to be even more important in EOL care settings [8, 27]. A study emphasized the importance of writing narratives about difficult encounters that promote interns’ reflection and self-awareness [28]. Another report suggests that dialogue with dying patients would enable residents to realize how wonderful life can be, to become aware of the importance of being a human before being a doctor, and, finally, to cultivate their humanity [29]. It has also been said that physicians value discussions about such cases with colleagues to become professional and reflective practitioners [24]. Therefore, attending physicians should utilize reflective discussions with team members, including residents, to improve the learning environment, to reduce stress on the medical team, and, ultimately, to enhance the care of patients and their families. Attending physicians should take these various approaches into account to promote deep reflection about residents’ experiences.

This study also clarified the conceptualization and the future planning process in ELT as well as the identity formation process and the related crises of residents in caring for dying patients. Clinical experience with dying patients or “caring for a person” caused the residents to reconstruct their identities as doctors and as future attending physicians while they were being affected by disruptions in their own emotions. Our study showed that the words and actions of the surrounding health professionals, including attending physicians, had a strong impact on the residents’ future. While they are learning about the duties of being a doctor, they are simultaneously establishing their identities as doctors [30, 31]. Newly qualified doctors experience an uncomfortable feeling that is caused by a gap between the responses to “*who am I?*” and “*what am I actually doing*?” Additionally, they are likely to experience "obstacles to integrating their identity into work" [30]. If there is a high degree of autonomy at work, individuals can develop their identity through participation in their duties. However, if the level of autonomy is limited, as in the case of residents, the formation of a professional identity can be disrupted [30]. Our study also indicated that the behavior of attending physicians discouraged residents and interfered with their identity formation. Thus, all attending physicians must respect the autonomy of residents and share responsibilities to develop their professional identity.

### Limitations

This study has several limitations. First, it was confined to interviews with residents who were working in acute phase hospitals in large urban areas. These settings differ from palliative care facilities in the narrow sense of the term, and many of the places in which these residents were completing their training are acute phase hospitals. This study seems to be significant because it obtained suggestions for learning and providing guidance in these types of settings. Second, this study focused specifically on Japan, which is part of Asia. Thus, the residents’ experiences, reflections, and processes of building awareness may be different from those in other cultural spheres. However, in the current situation, in which progress is being made in the global medical environment, there seems to be a fair amount of significance to understanding the characteristics of diverse cultural spheres and sharing each nation’s expertise. Third, the findings may not be generalizable due to the small number of participants. More studies should be conducted to cover various factors, including the participants’ personalities, previous learning experiences, perceptions of clinical learning, and the negative factors that influence residents’ emotional states. Further study on the perspectives from not only the residents but also the attending doctors and the nursing staff would also be necessary because many types of health care professions work together in EOL care.

## Conclusions

This qualitative study revealed Japanese residents’ perceptions, emotions and learning processes in caring for dying patients by applying Kolb’s ELT. The findings of this study may illuminate valuable pieces of knowledge for future education in EOL care.

## Abbreviations

ELT: experiential learning theory
EOL: end-of-life
